# *Mycoplasma pneumoniae* CARDS Toxin Exacerbates Ovalbumin-Induced Asthma-Like Inflammation in BALB/c Mice

**DOI:** 10.1371/journal.pone.0102613

**Published:** 2014-07-24

**Authors:** Jorge L. Medina, Jacqueline J. Coalson, Edward G. Brooks, Claude Jourdan Le Saux, Vicki T. Winter, Adriana Chaparro, Molly F. R. Principe, Laura Solis, T. R. Kannan, Joel B. Baseman, Peter H. Dube

**Affiliations:** 1 Department of Microbiology and Immunology, University of Texas Health Science Center at San Antonio, San Antonio, Texas, United States of America; 2 Department of Pathology, University of Texas Health Science Center at San Antonio, San Antonio, Texas, United States of America; 3 Department of Pediatrics, Division of Allergy and Infectious Diseases, University of Texas Health Science Center at San Antonio, San Antonio, Texas, United States of America; 4 Department of Medicine, Division of Cardiology, University of Texas Health Science Center at San Antonio, San Antonio, Texas, United States of America; 5 Center for Airway Inflammation Research, University of Texas Health Science Center at San Antonio, San Antonio, Texas, United States of America; National Jewish Health, United States of America

## Abstract

*Mycoplasma pneumoniae* causes a range of airway and extrapulmonary pathologies in humans. Clinically, *M. pneumoniae* is associated with acute exacerbations of human asthma and a worsening of experimentally induced asthma in mice. Recently, we demonstrated that Community Acquired Respiratory Distress Syndrome (CARDS) toxin, an ADP-ribosylating and vacuolating toxin synthesized by *M. pneumoniae*, is sufficient to induce an asthma-like disease in BALB/cJ mice. To test the potential of CARDS toxin to exacerbate preexisting asthma, we examined inflammatory responses to recombinant CARDS toxin in an ovalbumin (OVA) murine model of asthma. Differences in pulmonary inflammatory responses between treatment groups were analyzed by histology, cell differentials and changes in cytokine and chemokine concentrations. Additionally, assessments of airway hyperreactivity were evaluated through direct pulmonary function measurements. Analysis of histology revealed exaggerated cellular inflammation with a strong eosinophilic component in the CARDS toxin-treated group. Heightened T-helper type-2 inflammatory responses were evidenced by increased expression of IL-4, IL-13, CCL17 and CCL22 corresponding with increased airway hyperreactivity in the CARDS toxin-treated mice. These data demonstrate that CARDS toxin can be a causal factor in the worsening of experimental allergic asthma, highlighting the potential importance of CARDS toxin in the etiology and exacerbation of human asthma.

## Introduction

Asthma is a chronic disease characterized by reversible airway obstruction, inflammation, and airway hyperreactivity (AHR) [1]. While the causes of asthma are multi-factorial, asthma exacerbations can be triggered by viral or bacterial infections leading to greater morbidity [2], [3], [4], [5], [6], [7], [8]. *Mycoplasma pneumoniae* is a common human bacterial pathogen that causes acute or chronic respiratory infections and is linked to a variety of extrapulmonary infections or sequelae [2], [9], [10]. Clinical associations of *M. pneumoniae* infection with asthma exacerbations are increasing as detection methods improve [5], [7], [11], [12], [13], [14], [15], but the molecular mechanisms by which *M. pneumoniae* worsens asthma in humans are not well understood.

Experimental infections of mice with *M. pneumoniae* can mimic many aspects of *M. pneumoniae*-associated pathologies observed during human infection. For example, acute infection with *M. pneumoniae* can lead to pneumonia, increased lymphocytic inflammation, and AHR [11], [16]. Additionally, *M. pneumoniae* infection and its impact on pre-existing allergic inflammation have been tested in mouse models of asthma. These studies demonstrate the capacity of *M. pneumoniae* to exacerbate many features associated with allergic inflammation including T-helper type 2 (Th2) responses and AHR [17], [18].

Recently, we characterized a *M. pneumoniae*-encoded toxin capable of producing an asthma-like disease in naïve mice [19], [20], [21], [22]. This toxin designated Community Acquired Respiratory Distress Syndrome (CARDS) toxin, has vacuolating and ADP-ribosylating properties causing cytopathic effects both *in vitro* and *in vivo* [20]. We showed that recombinant CARDS toxin (rCARDS toxin) produces a robust lymphocytic and eosinophilic inflammation, leading to an asthma-like disease in naïve mice. rCARDS toxin-induced inflammation is characterized by pulmonary eosinophilia, increased expression of Th2 cytokines and chemokines, mucus metaplasia, AHR, and dependence on CD4^+^ cells consistent with allergic inflammation [22]. Importantly, unlike most mouse models of asthma, this study demonstrated the ability of rCARDS toxin to cause asthma-like disease in naïve mice after a single toxin exposure. Altogether, these data suggest that CARDS toxin is a potent inducer of allergic-type inflammation in mice.

The strongest clinical link between *M. pneumoniae* and asthma is in the acute exacerbation of asthma, with several recent studies demonstrating this correlation [6], [14], [15], [23], [24], [25]. For example, we reported that *M. pneumoniae* was detected in 52% of the respiratory secretions from a cohort of refractory asthmatics. Among the *M. pneumoniae*-positive refractory asthmatic patients, all tested positive for CARDS toxin by antigen capture indicating the presence of the toxin in the airway and suggesting an important role of CARDS toxin in asthmatic lung disease [14]. Additionally, Wood et al showed a strong correlation between poor asthma control and testing positive for CARDS toxin by antigen capture suggesting that *M. pneumoniae* infection and CARDS toxin can worsen asthma symptom severity and control [15]. In the current study, we evaluated the impact of rCARDS toxin on exacerbations of acute asthmatic responses using the OVA mouse model of asthma.

## Materials and Methods

### Ethics statement

This study was performed in accordance with animal use protocols approved by the University of Texas Health Science Center at San Antonio (UTHSCSA) Institutional Animal Care and Use Committee.

### Animals

5 week old BALB/cJ mice were purchased from Jackson Laboratory (Bar Harbor, ME) and maintained in an AAALAC-approved facility in accordance with Institutional Biosafety Committee and Institutional Animal Care and Use Committee protocols established at UTHSCSA.

### Recombinant CARDS toxin

rCARDS toxin was expressed and purified as previously described in detail [20], [21] and bioactivity was assessed by its ability to induce vacuoles in HeLa cells [19], [20]. The rCARDS toxin carrier fluid (CF) (filter sterilized 50 mM tris buffer with 5% glycerol at pH 7.3) was used as a vehicle control.

### OVA treatment and exposure to rCARDS toxin

Mice were sensitized and challenged with OVA using a modified protocol previously described [26]. Briefly, aluminum hydroxide solution (Alum) (Sigma, St. Louis, MO) was diluted in saline to 25% vol:vol and mixed with OVA overnight. 20 µg of OVA adsorbed to Alum in a volume of 100 µL were injected intraperitoneally twice, 2 weeks apart. Mice were subsequently challenged 2 weeks after the last injection with 1% OVA in saline by nebulization for 20 minutes daily for three days. Mice were rested 48 hours prior to intranasal or intratracheal instillation of 700 pmol of rCARDS toxin (OVA + rCARDS toxin group) or CF (OVA group). There were no statistically significant differences detected between instillation protocols (data not shown). Data acquisition was performed 7 days after CARDS toxin treatment, at the peak of CARDS toxin-induced inflammation.

### Bronchoalveolar lavage fluid (BALF) and cellular differentials

BALF was obtained as previously described [19], [27]. Cells in the BALF were washed and counted before centrifugation onto microscope slides using a cytospin 2 centrifuge (Shandon; Thermo, Waltham, MA). Slides were stained with Wright-Giemsa based stain (Diff-stain; IMEB Inc, San Marcos, CA), and relative numbers of neutrophils, eosinophils, monocytes/macrophages, and lymphocytes were counted.

### Cytokine analysis

Enzyme-linked immunosorbent assays (ELISA) were used to determine concentrations of eotaxin-1 and 2, CCL17 and CCL22 in BALF samples according to manufacturer's instructions (R&D Systems, Minneapolis, MN).

### Quantitative real-time PCR (qRT-PCR)

RNA was isolated from the lungs of OVA + rCARDS toxin or OVA mice 7 days after rCARDS toxin or CF exposure, using Life Technologies Trizol reagent according to manufacturer's protocols. RNA quality and purity were determined spectrophotometrically; all RNA absorbance ratios were between 1.9 and 2.2. Total RNA was reverse transcribed and subjected to PCR with SYBR-green using an Applied Biosystem's 7900HT thermal cycler. Relative changes in mRNA expression were determined by the ^ΔΔ^CT method using actin normalization. The following primer pairs were used: 5′-3′ actin forward tggaatcctgtggcatccatgaaac; actin reverse aaaacgcagctcagtaacagtccg; CCL17 forward atgccagagctgctcgag; CCL17 reverse tgccctggacagtcagaaac; CCL22 forward ggtccctatggtgccaatgt; CCL22 reverse acggatgtagtcctggcagc; IL-4 forward cagcaacgaagaacaccacag; IL-4 reverse ccttggaagccctacagacg and IL-13 forward tcacacaagaccagactcccc; IL-13 reverse ccacactccataccatgctgc.

### Histopathology and immunohistochemistry (IHC)

Following instillation of rCARDS toxin, lungs were harvested 7 days after exposure. Tissues were fixed in 10% formalin solution and embedded in paraffin, from which 4 µm sections were cut and stained with hematoxylin and eosin (H&E). Eosinophils were detected using a monoclonal antibody against eosinophil major basic protein (MBP) Clone MT-14.7 (Lee Laboratory, Mayo Clinic, Arizona) and evaluated by digital pathology using Aperio Scanscope XT (Aperio, Vista CA). Complete images of lungs were obtained digitally using the Aperio Scanscope XT. All slides were evaluated by a pulmonary pathologist, J.C., who was blinded to toxin-treated and control groups. Slides representative of the spectrum of inflammatory responses were identified for use in pathology scoring analysis as previously described [28], [29]. The extent of gross histological changes between experimental groups was evaluated by 4 individuals using the panel of standards, with grade 1 being the least severe and grade 3 the most severe [22], [28], [29]. Images of the graded standards are presented as Fig S1A-C.

### Airway function

Changes in pulmonary function of OVA + rCARDS toxin or OVA mice were assessed using the Flexivent system (SCIREQ, Montréal, Canada) and Flexivent software version 5.2. Pulmonary function parameters of dynamic resistance and dynamic compliance were measured through a dose-response challenge of aerosolized methacholine 7 days after rCARDS toxin instillation as we described previously [22].

### Statistical analysis

Experiments were repeated a minimum of 2 times with 3-16 animals, depending on the specific protocol. All results were expressed as the mean +− S.D. Statistical differences were determined using a two-way ANOVA, two-tailed Student *t-*test, or Mann-Whitney U test for normally or non-normally distributed data with GraphPad Prism version 5.04 (GraphPad Software, San Diego CA). Fleiss' kappa test was used to measure agreement within multiple observers' analysis of the panel of standards [30], [31]. A *p* value of <0.05 was considered significant.

## Results

### rCARDS toxin exacerbates cellular inflammation in OVA model of asthma

**Figure 1:**
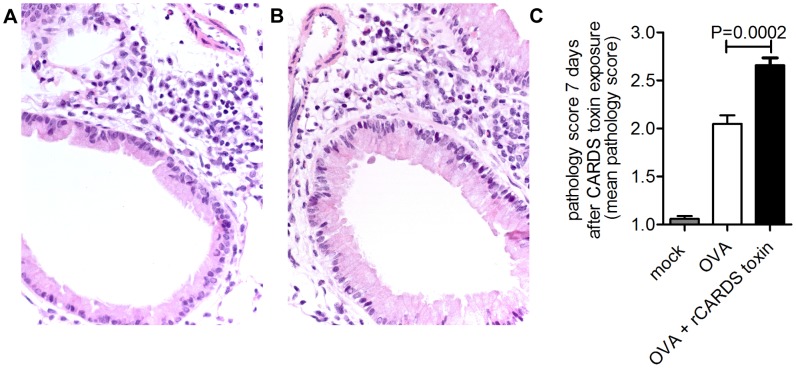
rCARDS toxin exacerbates inflammation associated with OVA treatment. A-B) H&E stained lung sections demonstrating inflammation in OVA (panel A) or OVA + rCARDS toxin (panel B) 40x magnification. C) Quantification of average histological scores from OVA or OVA + rCARDS toxin mice on day 7 (p = 0.0002).

We have shown that exposure of naïve BALB/cJ mice to rCARDS toxin resulted in an eosinophilic-rich peribronchiolar and perivascular cellular inflammation, resembling asthma [19], [22]. To test the capacity of CARDS toxin to exacerbate preexisting asthma-like inflammation, BALB/cJ mice were treated with OVA and challenged with rCARDS toxin 48 hours after the last OVA challenge, at the peak of OVA-induced allergic inflammation. Lungs were harvested and analyzed 7 days after rCARDS toxin exposure. H&E-stained lung sections revealed increased cellular inflammation in OVA or OVA + rCARDS toxin relative to mock-treated controls (Fig. 1A-B). Animals treated with OVA + rCARDS toxin had a higher average pathological score compared to OVA mice at day 7 (2.65+−0.30 vs. 2.05+−0.33 respectively (p = 0.0002)) suggesting an enhancement of cellular inflammation in the rCARDS toxin-treated mice (Fig 1C). Determination of agreement between investigators blinded to the treatment groups was performed using Fleiss' kappa test [30], [31]. This analysis determined a value of Κ = 0.67 indicating substantial agreement amongst the investigators. In total, these data suggest rCARDS toxin is a causal factor in exacerbation of gross histological changes observed in OVA-induced experimental asthma.

### rCARDS toxin exacerbates eosinophilic inflammation in OVA model of asthma

**Figure 2:**
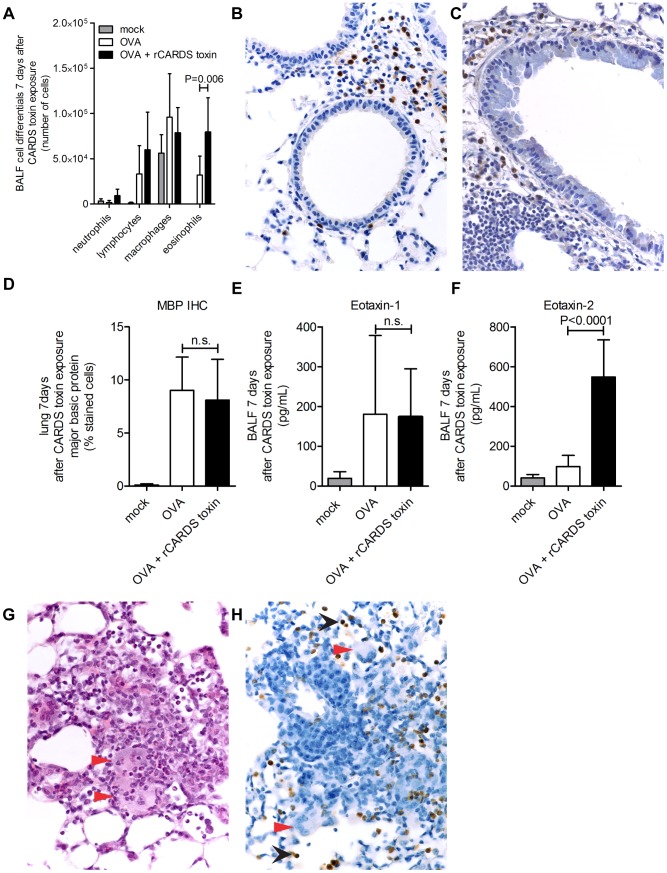
rCARDS toxin exacerbates eosinophilia. A) Cellular differentials from BALF on day 7 after exposure to CF or rCARDS toxin. Increased eosinophilia is seen at day 7 (p = 0.006). B-C) Eosinophils at peribronchiolar lesions of OVA (B) or OVA + rCARDS toxin (C) detected by immunohistochemistry with anti-MBP. Brown color depicts positive staining for eosinophil MBP. Original magnification 40x. D) Quantification of bronchiolar MBP immunohistochemistry stain using Aperio digital pathology system. E) Measurement of eotaxin-1 concentration by ELISA (n.s.). F) Differences in eotaxin-2 concentrations determined by ELISA (p<0.0001). G) H&E stain depicting eosinophil-rich granulomas 40x magnification. Red arrows point towards giant cells. H) MBP immunohistochemistry of granulomatous lesion 40x magnification. Black arrows point towards eosinophils and red arrows point towards giant cells.

Previously, we demonstrated that rCARDS toxin alone induces significant eosinophilia in BALF and lung parenchyma of naïve mice [22]. Additionally, the OVA mouse model of asthma reported to be a well-defined model of allergic airway inflammation that induces significant eosinophilia [32]. We tested the potential of rCARDS toxin to exacerbate eosinophilia in the OVA model of asthma. Mice were analyzed for eosinophil infiltration 7 days after rCARDS or CF treatments, by analyzing BALF, cellular differentials, and histological assessments of lung tissue. OVA + rCARDS toxin-treated mice had significantly increased numbers of eosinophils in the BALF compared to mice treated with OVA alone, (79,500+−37,792 vs. 31,933+−20,941 eosinophils (p = 0.0063)) respectively (Fig. 2A). Peribronchiolar inflammatory lesions in the OVA + rCARDS toxin-treated mice had equivalent eosinophil infiltration compared to OVA mice as analyzed by digital pathology (Fig. 2B-D).

It was reported that the eosinophil chemokine, eotaxin-2, preferentially draws eosinophils into the airway luminal spaces [33]. To test if the increased infiltration of eosinophils into the BALF correlated with differential expression of eotaxin-2, BALF samples from animals in the different treatment groups were analyzed for the presence of eotaxin-1 and 2. Only eotaxin-2 was found to be significantly different between experimental groups, OVA vs. OVA + CARDS, (98.5+−55.6 pg/mL vs 548.2+−186.6 pg/mL (p<0.0001)) respectively (Fig. 2F), whereas there was equivalent expression of eotaxin-1 protein in the lungs of OVA and OVA + rCARDS toxin treated mice (Fig. 2E). These data suggest rCARDS toxin treatment of mice with experimentally induced asthma is able to increase expression of eotaxin-2 and worsen airway eosinophilia.

A histological feature sometimes observed with mouse models of chronic asthma is the development of parenchymal inflammation which can be granulomatous in nature [34]. Although we were using acute OVA sensitization, features of chronic inflammation, including the presence of granulomas, were seen when OVA-treated mice were exposed to rCARDS toxin (Fig. 2G-H). These granulomas were rich in eosinophils, a finding also observed, to a lesser extent, in naïve animals treated with rCARDS toxin (unpublished data). Collectively, these data suggest that rCARDS toxin exacerbates OVA-induced allergic inflammation and eosinophilia.

### rCARDS toxin exposure leads to increased pulmonary expression of Th2 cytokines and chemokines in OVA-treated mice

**Figure 3:**
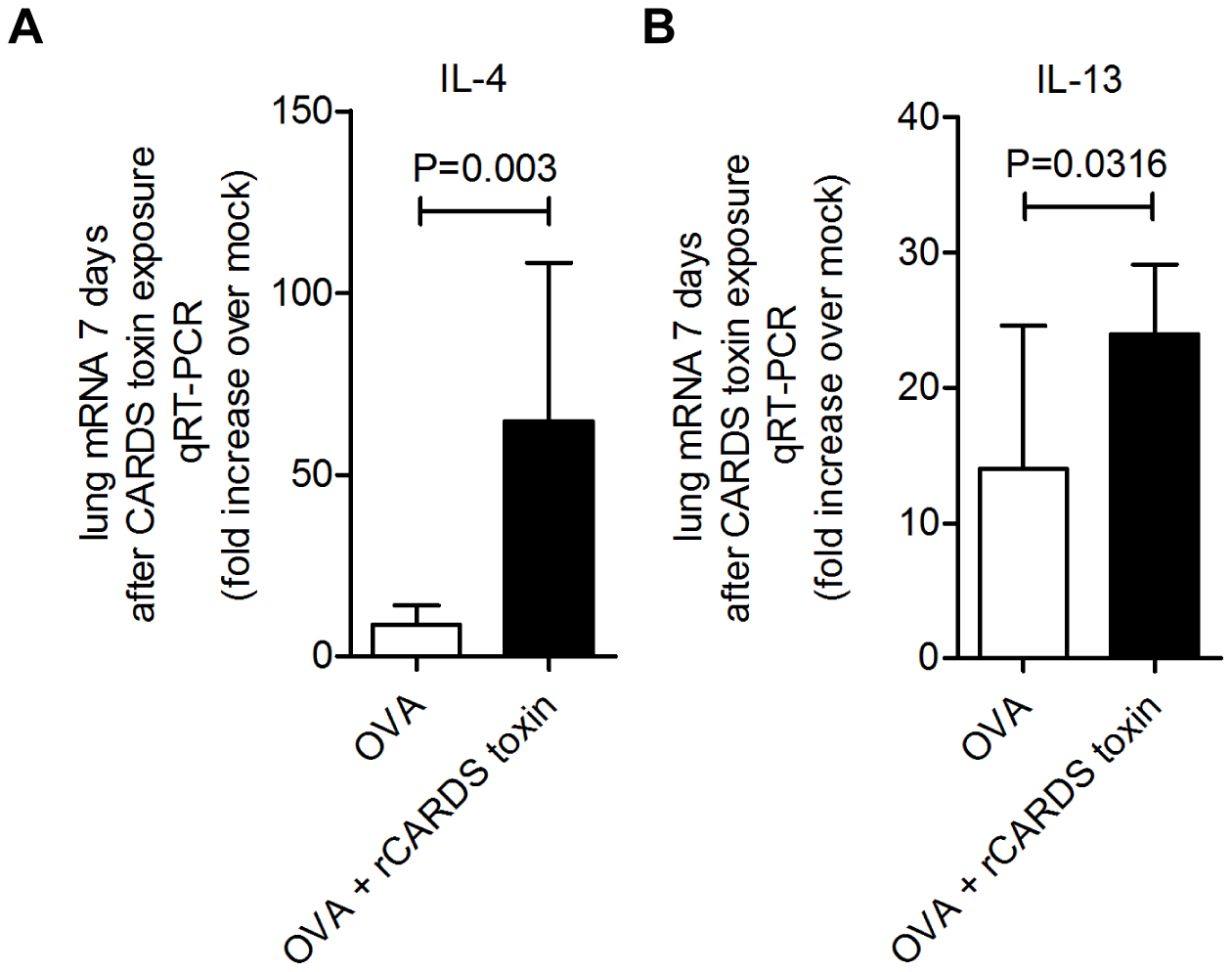
rCARDS toxin increases the induction of Th2 effector cytokines in OVA treated mice. RNA was extracted from lungs of OVA- or OVA + rCARDS toxin-treated mice. qRT-PCR quantification of mRNA induction for A) IL-4 (p = 0.003) and B) IL-13 (p = 0.0316) 7 days after exposure.

We demonstrated that rCARDS toxin exposure induces expression of Th2 cytokines in naïve BALB/cJ mice [22]. IL-4 and IL-13 are two central Th2 effector cytokines upregulated in OVA-induced allergic asthma [35]. To evaluate the impact of CARDS toxin on the expression of Th2 cytokines in a preexisting asthma model, lungs were analyzed for IL-4 and IL-13 mRNA 7 days after exposure to rCARDS toxin and compared to mock-treated controls. We observed approximately 8-fold (p = 0.003) and 1.7-fold increases (p = 0.032) in IL-4 and IL-13 mRNA expression, respectively, in OVA + rCARDS toxin-exposed mice compared to OVA alone (Fig. 3A & B).

**Figure 4:**
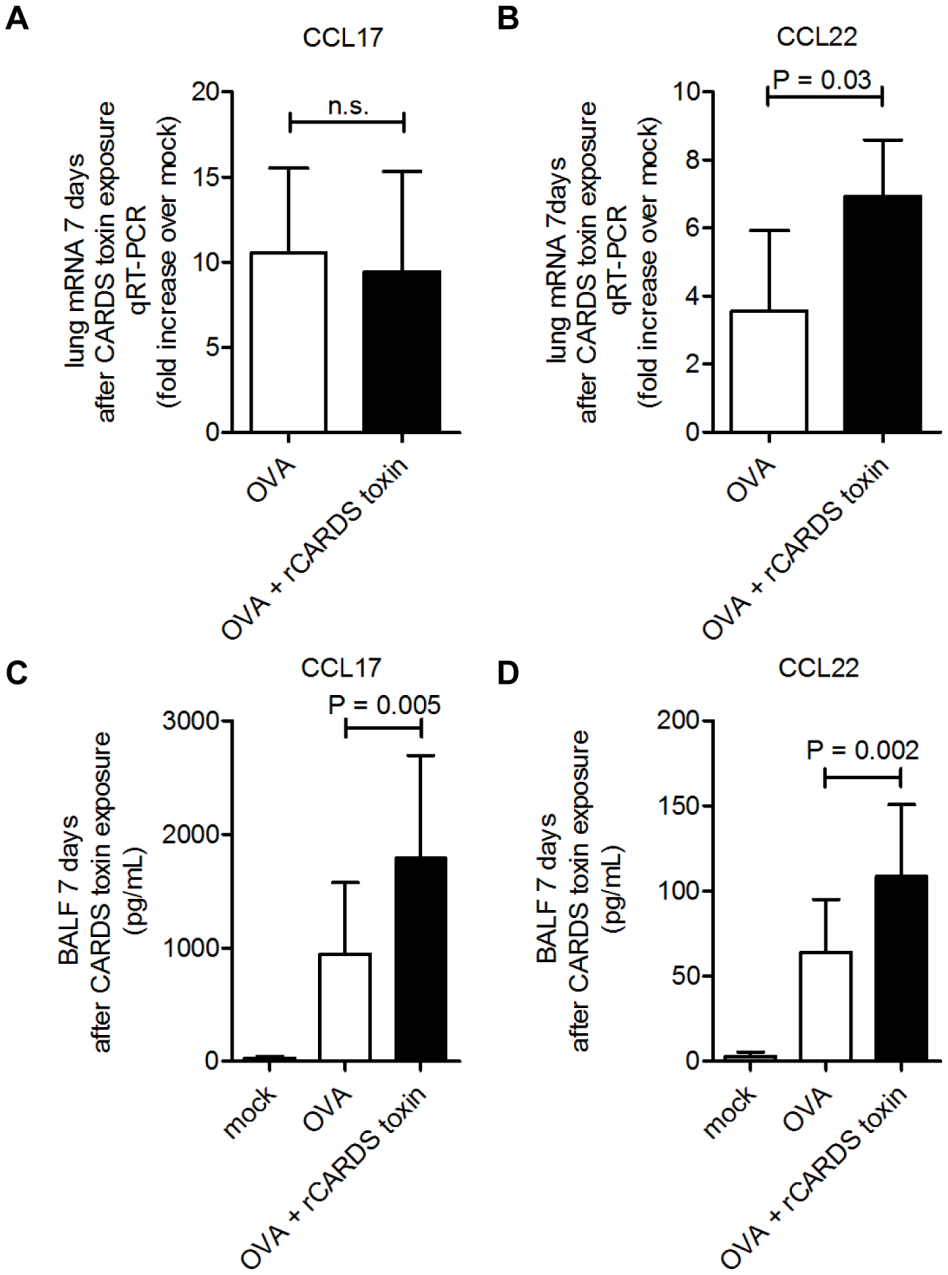
Chemokines CCL17 and CCL22 expression is increased in OVA-treated mice exposed to rCARDS toxin. qRT-PCR quantification of mRNA induction for A) CCL17 and B) CCL22 (p = 0.03) 7 days after exposure. Chemokine concentrations were measured by ELISA for C) CCL17. (p = 0.005) and D) CCL22 (p = 0.002).

The chemokines CCL17 and CCL22 recruit Th2 T-cells to sites of inflammation [36]. However, lung CCL17 mRNA expression 7 days after CARDS toxin exposure was not significantly different in OVA + rCARDS toxin mice when compared to OVA mice (Fig. 4A). In contrast, there were significantly increased concentrations of CCL17 protein in OVA + rCARDS toxin-treated mice compared to OVA mice (1,791+−907.6 pg/mL vs 944.9+−632.7 pg/mL, respectively (p = 0.005)) (Fig. 4C). CCL22 mRNA expression was increased 2-fold (p = 0.03) (Fig. 4B), and CCL22 protein concentrations were increased in the BALF for OVA + rCARDS toxin vs OVA mice (108.5+−42.4 pg/mL vs 64.1+−30.8 pg/mL, respectively (p = 0.002)) (Fig. 4D). In total, these data suggest that Th2 cytokine and chemokine expression is heightened by rCARDS toxin exposure in a model of preexisting allergic inflammation, contributing to exacerbation of Th2-mediated inflammation.

### rCARDS toxin exacerbates airway hyperreactivity in OVA-treated mice

**Figure 5:**
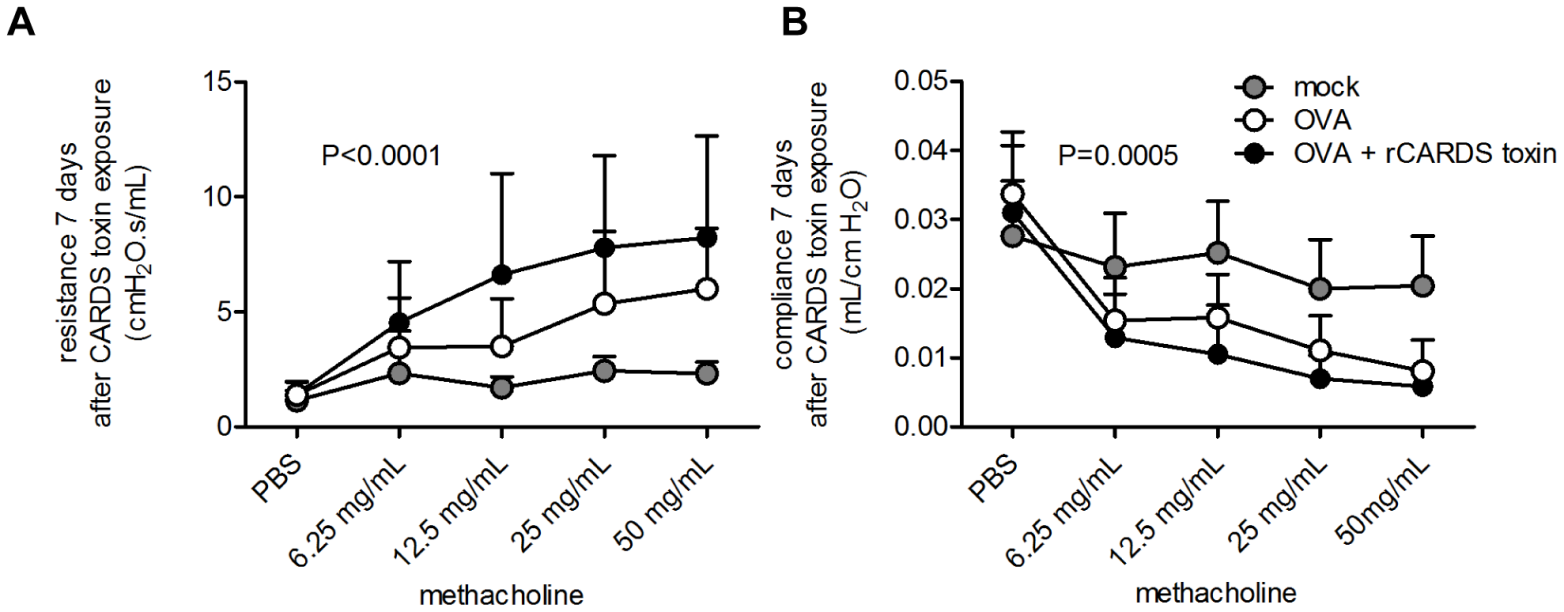
rCARDS toxin exacerbates OVA-induced airway hyperreactivity in BALB/cJ mice. Airway hyperreactivity was directly measured by changes in airway resistance and lung compliance through a methacholine dose curve. (A) Dynamic airway resistance (p<0.0001) and (B) Dynamic compliance (p = 0.0005) measurements were made 7 days after CARDS toxin exposure and analyzed using 2-way ANOVA.

Because rCARDS toxin induces AHR in naïve mice, we evaluated the effects of rCARDS toxin on pre-existing hyperreactive airways in the OVA model of asthma. The airway mechanics of OVA or OVA + rCARDS toxin mice were measured via direct pulmonary function 7 days after exposure to rCARDS toxin. As shown in Figure 5 A-B, there was a significant increase in airway resistance (p<0.0001) and reduction in lung compliance (p = 0.0005) associated with rCARDS toxin exposure in mice with pre-existing allergic inflammation on day 7, indicative of worsened AHR. These data demonstrate that rCARDS toxin is capable of enhancing preexisting airway obstruction and exacerbating AHR in the mouse model of allergic asthma.

## Discussion

*Mycoplasma pneumoniae* is a common human atypical bacterial pathogen that causes up to 40% of community acquired pneumonias and is implicated in exacerbations of human asthma [9]. For example, Lieberman et al used serology to demonstrate acute infection with *M. pneumoniae* in 18% of patients with asthma exacerbation compared to 3% of a matched control group [6]. Likewise, it was shown that chronic, stable asthma patients who tested positive for *M. pneumoniae* or *Chlamydia pneumoniae* by PCR and were treated with clarithromycin experienced an improvement in airway function, while patients who tested negative for *M. pneumoniae* or *C. pneumoniae* experienced no improvement suggesting a role for these bacteria in disease pathogenesis [5]. While there are many clinical studies implicating *M. pneumoniae* in exacerbations of asthma, the mechanisms responsible for exacerbation of disease are not fully understood [6], [14], [15], [23], [24], [25].

We demonstrated that rCARDS toxin, an ADP-ribosylating and vacuolating toxin, is sufficient to cause allergic-type inflammation in naïve BALB/cJ mice [22], [37]. In the current study, we investigated the impact of rCARDS toxin in exacerbation of allergic inflammation. Exposure to rCARDS toxin was sufficient to significantly increase pulmonary allergic inflammation as manifested by increased cellular inflammation in mice with preexisting OVA-generated experimental asthma. CARDS toxin-mediated exacerbation of disease was accompanied by increased expression of Th2-mediated cytokine and chemokine responses and enhanced AHR.

The inflammation associated with the OVA model of asthma is characterized by the influx of eosinophils and Th2 T-cells and epithelial cell hyperplasia, and is similar in many respects to human allergic asthma. Our findings suggest that CARDS toxin is a significant virulence factor in the exacerbations of OVA-induced inflammation in mice infected with live *M. pneumoniae*, as mycoplasmas synthesize augmented levels of CARDS toxin in the airways [38] and rCARDS toxin alone recapitulates the exaggerated pathologies observed during *M. pneumoniae* infection of OVA-treated mice [17], [39]. These data strongly suggest that the exacerbation of allergic inflammation observed with *M. pneumoniae* infection of OVA-treated mice was due in part to the actions of the CARDS toxin. In addition, CARDS toxin is readily detected in *M. pneumoniae*-infected humans and mice [14], [15], [40]. Previous studies evaluating the potential of *M. pneumoniae* to exacerbate OVA-induced models of asthma demonstrated increased expression of Th2 cytokines and pronounced eosinophilia [18], [39]. These studies highlighted the importance of understanding *M. pneumoniae* as a human pathogen, but did not identify a virulence factor(s) responsible for exacerbation of allergic inflammation in OVA models. Our data implicate CARDS toxin as a major virulence factor contributing to the exaggerated inflammatory responses observed when OVA-treated mice are exposed to rCARDS toxin.

rCARDS toxin and OVA treatments are both known to induce a significant eosinophilic inflammation in the lungs of mice [22], [32]. Additionally, increased numbers of eosinophils correlated with more severe forms of asthma in humans, such as refractory eosinophilic asthma [41], [42], [43]. The contributions of eosinophils to the pathology of asthma are not entirely clear, and newer data suggest that these cells are more complex than previously thought [36]. Eosinophils are able to express a variety of inflammatory mediators such as MBP, eosinophil peroxidase, leukotrienes and cytokines, such as IL-4 and IL-13, consistent with the role of eosinophils in allergic inflammation [41]. Eosinophil-derived inflammatory mediators have significant impact on bronchiolar epithelium and airway smooth muscle hyperresponsiveness. In mice, eosinophils are one of the initial cells responding to allergic inflammation, where they can act as antigen-presenting cells and contribute to the recruitment of T-cells to sites of inflammation [44]. The specific role of eosinophils was not investigated in this current study, but we observed increased numbers of eosinophils in the BALF and increased severity of inflammation. Therefore, it is reasonable to consider eosinophils as contributing to the pathology of CARDS toxin-associated exacerbations of asthma in mice. Granulomas with an eosinophilic component were noted in the OVA + rCARDS-treated mice and resembled a human asthmatic pathology, asthmatic granulomatosis, recently observed in severe asthma [45]. Although this is an enticing correlate, it remains unknown if CARDS toxin has a role in asthmatic granulomatosis associated with severe asthma in humans.

Th2 responses and inflammation have direct effects on AHR and airway obstruction. In this study we demonstrate that rCARDS toxin is sufficient to exacerbate these responses in a mouse model of asthma. Recent clinical studies linking *M. pneumoniae* infections with acute exacerbations of asthma detected CARDS toxin in patients, suggesting that CARDS toxin could have a similar impact on human health. CARDS toxin is readily detected in the respiratory secretions of subsets of both children and adults with refractory asthma or acute exacerbations of asthma [14], [15]. In these patients, CARDS toxin is measured by PCR or antigen capture in 45% of subjects with refractory asthma compared to 16.5% of normal controls. The importance of these correlations was highlighted by the observation that after 8 weeks of macrolide treatment, a patient's asthma symptoms failed to improve, and CARDS toxin was persistently detected in the sputum of the macrolide-treated patient [14]. Additionally, our recent data show that children with acute or refractory asthma that test positive for *M. pneumoniae* by PCR or CARDS toxin antigen capture, have lower exhaled breath condensate pH, lower quality of life scores, and are less likely to have their asthma under control [15]. Taken together, these data suggest that *M. pneumoniae* and CARDS toxin could impact disease severity in asthmatics.

Data presented in the current study demonstrate increased severity of experimentally induced asthma-associated lung pathologies in BALB/cJ mice following exposure to rCARDS toxin compared to OVA treatment alone. Altogether, these data suggest that CARDS toxin exacerbates OVA-induced asthma in mice [17], [18], [39]. The identification of CARDS toxin as a key *M. pneumoniae* virulence factor that enhances experimental asthma in mice will facilitate understanding the role of *M. pneumoniae* in human asthma. These findings warrant further studies to understand the impact of CARDS toxin as a mediator in the exacerbations of human asthma during *M. pneumoniae* infections.

## Supporting Information

Figure S1 **Panel of standards used in evaluating inflammatory lung pathology.** Whole lung H&E sections were evaluated by 4 individuals blinded to experimental treatments. (A-C) Pathology scores of 1-3 were assigned using a panel standards (A = 1, B = 2, C = 3).
(TIF)

Figure S2 **Representative H&E image from a lung section of a mock treated animal (40x).**
(TIF)
