# Supplementary figures and images for: Mycoplasma pneumoniae CARDS Toxin Exacerbates Ovalbumin-Induced Asthma-Like Inflammation in BALB/c Mice
Source: PLoS One. 2014 Jul 24;9(7):e102613. doi: 10.1371/journal.pone.0102613 (PMC4109942; doi:10.1371/journal.pone.0102613)

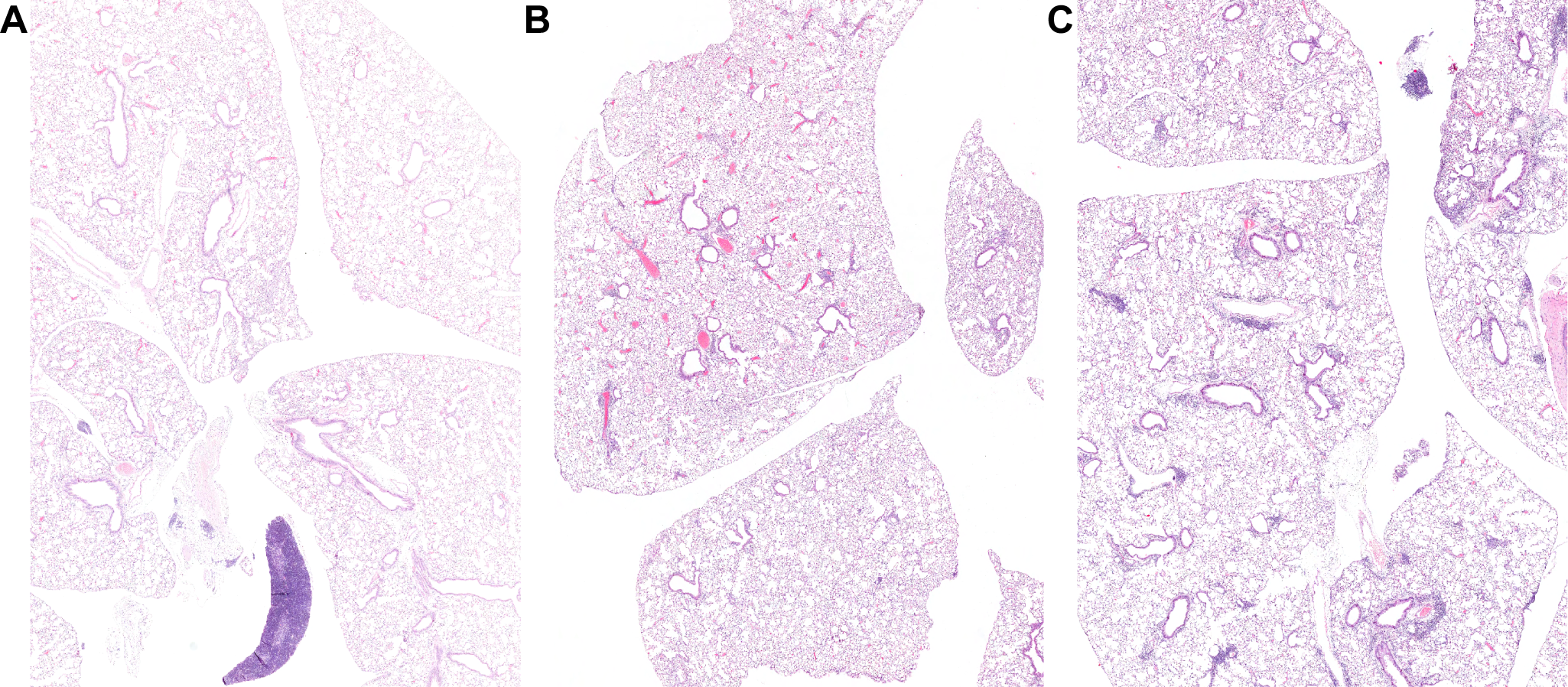

Supplement: Figure S1 — Panel of standards used in evaluating inflammatory lung pathology. Whole lung H&E sections were evaluated by 4 individuals blinded to experimental treatments. (A-C) Pathology scores of 1-3 were assigned using a panel standards (A = 1, B = 2, C = 3). (TIF) [file pone.0102613.s001.tif]

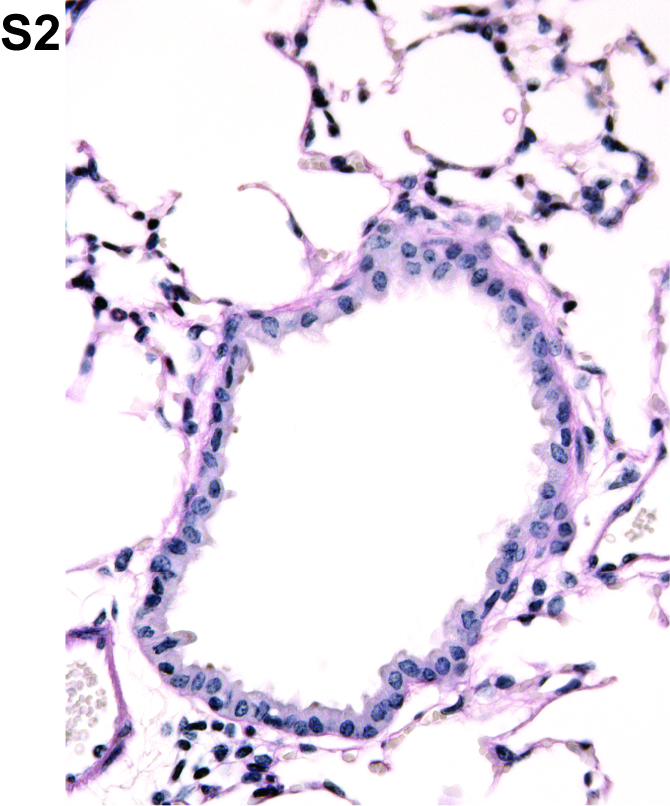

Supplement: Figure S2 — Representative H&E image from a lung section of a mock treated animal (40x). (TIF) [file pone.0102613.s002.tif]
